# The role of the bronchial microvasculature in the airway remodelling in asthma and COPD

**DOI:** 10.1186/1465-9921-11-132

**Published:** 2010-09-29

**Authors:** Andrea Zanini, Alfredo Chetta, Andrea S Imperatori, Antonio Spanevello, Dario Olivieri

**Affiliations:** 1 Salvatore Maugeri Foundation, Department of Pneumology, IRCCS Rehabilitation Institute of Tradate, Italy; 2 Department of Clinical Sciences, Section of Respiratory Diseases, University of Parma, Italy; 3 Center for Thoracic Surgery, University of Insubria, Varese, Italy; 4 Department of Respiratory Disease, University of Insubria, Varese, Italy

## Abstract

In recent years, there has been increased interest in the vascular component of airway remodelling in chronic bronchial inflammation, such as asthma and COPD, and in its role in the progression of disease. In particular, the bronchial mucosa in asthmatics is more vascularised, showing a higher number and dimension of vessels and vascular area. Recently, insight has been obtained regarding the pivotal role of vascular endothelial growth factor (VEGF) in promoting vascular remodelling and angiogenesis. Many studies, conducted on biopsies, induced sputum or BAL, have shown the involvement of VEGF and its receptors in the vascular remodelling processes. Presumably, the vascular component of airway remodelling is a complex multi-step phenomenon involving several mediators. Among the common asthma and COPD medications, only inhaled corticosteroids have demonstrated a real ability to reverse all aspects of vascular remodelling. The aim of this review was to analyze the morphological aspects of the vascular component of airway remodelling and the possible mechanisms involved in asthma and COPD. We also focused on the functional and therapeutic implications of the bronchial microvascular changes in asthma and COPD.

## Introduction

Bronchial vessels usually originate from the aorta or intercostal arteries, entering the lung at the hilum, branching at the mainstem bronchus to supply the lower trachea, extrapulmonary airways, and supporting structures. They cover the entire length of the bronchial tree as far as the terminal bronchioles, where they anastomose with the pulmonary vessels. The bronchial vessels also anastomose with each other to form a double capillary plexus. The external plexus, situated in the adventitial space between the muscle layer and the surrounding lung parenchyma, includes venules and sinuses, and it constitutes a capacitance system. The internal plexus, located in the subepithelial lamina propria, between the muscularis and the epithelium, is essentially represented by capillaries. These networks of vessels are connected by short venous radicles, which pass through the muscle layer structure. The bronchial submucosal and adventitial venules drain into the bronchial veins which drain into the azygos and hemiazygos veins [1-3].

In normal airways, the bronchial microvasculature serves important functions essential for maintaining homeostasis. In particular, it provides oxygen and nutrients, regulates temperature and humidification of inspired air, as well as being the primary portal of the immune response to inspired organisms and antigens [4]. The high density of capillaries present is probably related to a high metabolic rate in the airway epithelium, which is very active in secretory processes. In fact, the oxygen consumption of airway epithelium is comparable to that of the liver and the heart [1]. In normal airways, the maintenance of vascular homeostasis is the result of a complicated interaction between numerous pro- and anti-angiogenetic factors (Table 1). Bronchial flow may be affected by alveolar pressure and lung volume, with higher airway pressures decreasing blood flow [5]. Moreover, the bronchial arteries have α- and β-adrenergic receptors and it is known that adrenalin, which has α-agonist effects, reduces total bronchial flow as it does in other systemic vascular beds [6]. Lastly, vagus stimulation may increase total bronchial flow [5].

**Table 1:** Inducers and inhibitors of angiogenesis

Angiogenetic inducers	Angiogenetic inhibitors
**Inflammatory mediators**	**Soluble mediators**
IL-3, IL-4, Il-5, IL-8, IL-9, IL-13	IFN-α, IFN-β, IFN-γ
TNFα	Ang-2
Prostaglandin E_1_, E_2_	TIMP-1, TIMP-2
**Growth Factors**	IL-4, IL-12, IL-18
VEGF	Troponin
FGF-1, FGF-2	VEGI
PDGF	TSP-1, TSP-2
PIGF	PF-4
IGF	**Protein fragments**
TGFα, TGFβ	Angiostatin
EGF	Endostatin
HGF	aaAT
HIF	Prolactin
PD-ECGF	Vasostatin
**Enzymes**	**Tumor suppressor genes**
COX-2	P53
Angiogenin	NF1, NF2
MMPs	RB1
**Hormones**	DCC
Estrogens	WT1
Gonadotropins	VHL
TSH	
Proliferin	
**Oligosaccharides**	
Hyaluronan	
Gangliosides	
**Cell adhesion molecules**	
VCAM-1	
E-selectin	
α_v_β_3_	
**Hematopoietic factors**	
GM-CSF	
Erythropoietin	
**Others**	
Nitric oxide	
Ang-1	

During chronic inflammation, the vascular remodelling processes are the consequence of the prevalence of a pro-angiogenetic action, in which many growth factors and inflammatory mediators are involved [7]. Accordingly, the bronchial microvasculature can be modified by a variety of pulmonary and airway diseases. Congestion of the bronchial vasculature may narrow the airway lumen in inflammatory diseases, and the formation of new bronchial vessels, angiogenesis, is implicated in the pathology of a variety of chronic inflammatory, infectious, and ischemic pulmonary diseases [3,4,8]. Bronchiectasis and chronic airway infections may be characterized by hypervascularity and neo-vascularisation of the airway walls [9]. Additionally, airway wall ischemia following lung transplantation can induce new vessel formation [9]. The remarkable ability of the bronchial microvasculature to undergo remodelling has also implications for disease pathogenesis

**Figure 1:**
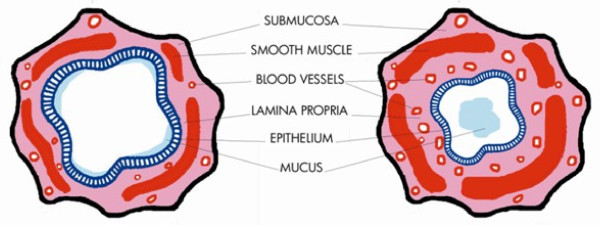
**Schematic picture of normal (left) and asthmatic airway (right), indicating the remodelling of compartments, with particular regards to microvascular alterations**.

Most of the literature regarding bronchial vascular remodelling in chronic airway inflammation results from studies in asthmatic patients [10-15], since the vascular component of airway remodelling significantly contributes to the alteration of the airway wall in asthma (Figure 1). Interestingly, it has been recently shown that bronchial vascular changes may also occur in COPD [16-18]. Microvascular changes in asthma and COPD may contribute to an increase in airway wall thickness which may be associated with disease progression [9]. This review focuses on the morphological aspects of the vascular component in airway wall remodelling in asthma and COPD and its functional and therapeutic implications.

## Asthma

Early observations regarding bronchial vascular changes in asthmatic airways date back to the sixties and concern post-mortem analysis [19,20]. Dunnill and colleagues showed oedematous bronchial mucosa with dilated and congested blood vessels in patients with fatal disease [19,20]. About thirty years later, some studies demonstrated an increase in the percentage of blood vessels in the airway walls; the concepts of vessel dilatation, increased permeability and angiogenesis were suggested [12,21].

### Hyperaemia and hyperperfusion

An increased blood flow has been shown in the airways of asthmatic patients, in comparison to healthy controls, by measuring dimethyl ether in exhaled air [22]. Calculated as the volume of the conducting airways from the trachea to the terminal bronchioles, mean airway blood flow values were found to be 24-77% higher in asthmatics than in healthy controls [22]. Increased blood flow is likely due to the dilatation of arterioles and an increased number of vessels.

Activation of pulmonary c-fibre receptors by irritants and inflammatory mediators may induce vasodilatation mainly via sympathetic motor nerves. Local axon reflexes in response to irritants and inflammatory mediators may release vasodilator neuropeptides such as substance P, neurokinins, and calcitonin gene-related peptides [23]. Consequently, the airway inflammation in asthma may evoke mucosal vasodilatation due to the direct action of mediators on vascular smooth muscle, neuropeptides released by axon reflexes from sensory nerve receptors, and reflex vasodilatation due to stimulation of sensory nerves [23]. Nitric oxide (NO) and blood flow regulation abnormalities by the sympathetic nervous system may be involved in hyperaemia and hyperperfusion, even if the precise mechanisms are unclear [24]. Notably, bronchial blood flow positively correlates with both exhaled nitric oxide NO and breath temperature in asthmatic subjects [25]. Exhaled breath temperature and bronchial blood flow may reflect rubor and calor in the airways, and therefore may be markers of tissue inflammation and remodelling [25].

### Microvascular permeability

Increased microvascular permeability and oedema are common features during vascular remodelling in bronchial asthma [26]. Electronic microscopy studies have shown that in the lower airways of most species, including healthy humans, only the capillaries underlying neuroepithelial bodies are fenestrated, the rest having a continuous epithelium [1]. By contrast, in some animal species the lower airway capillaries are fenestrated [27]. Interestingly, this feature is also observed in asthmatic patients [27]. Using animal models, McDonald et al [27] suggested a role for intercellular gaps between endothelial cells of postcapillary venules in microvascular permeability. Some reports confirmed the relevant presence of this phenomenon in airways of asthmatic patents [28-33]. In these studies, microvascular permeability was evaluated by the airway vascular permeability index as measured by the albumin in induced sputum/albumin in serum [28-31], or as fibronectin concentrations [32] or alpha 2-macroglobulin levels [33] in BAL fluid.

Plasma extravasations can compromise epithelial integrity and contribute to formation luminal mucus plugs [34]. Plasma leakage can also lead to mucosal oedema and bronchial wall thickening, thereby reducing the airway lumen, which in turn causes airflow limitation and may contribute to airway hyperresponsiveness [35,36]. Furthermore, during the chronic inflammation process, new capillaries are immature and unstable and can contribute to increased permeability [36]. The increased microvascular permeability is due to the release of inflammatory mediators, growth factors, neuropeptides, cytokines, eosinophil granule proteins, and proteases (Table 2).

**Table 2:** Factors involved in the bronchial vasculature remodelling in asthma and COPD

Angiogenesis	Vasodilatation	Permeability
VEGF	VEGF	VEGF
FGF	Histamine	Histamine
TGFβ	Heparine	Adenosin
HGF	Tryptase	Bradychinin
HIF	NO	Ang-1, Ang-2
Ang-1	TGFα, TGFβ	SP
Histamine	FGF	CGRP
PGD_2_	EGF	LTB_4_, LTC_4_, LTD_4_
PGI_2_	IL-4	PAF
LTC2	TNFα	ET-1
PAF	LTC_4_	TNFα
SP	PGD_2_	ECP
VIP		
IL-8, IL-13		
TNFα		
NKA		
Angiogenin		
MMPs		
IGF-1		
Chymase		
VCAM-1		
E-selectin		
α_v_β_3_		

Vascular endothelial growth factor (VEGF) is a potent angiogenic factor, proven to be increased in asthma and correlated to the vascular permeability of airways [28-30], as well as shown to determine gaps in the endothelium [37]. Angiopoietin-1 is known to stabilize nascent vessels, making them leak resistant, while Angiopoietin-2 reduces vascular integrity. Angiopoietin-2, but not angiopoietin-1, is positively correlated to the airway vascular permeability index [31]. Greiff et al [38] found that histamine was able to induce plasma extravasation with consequent bronchial exudation in healthy subjects. Similarly, bradykinin can determine plasma exudation in human peripheral airways. Berman et al [39] performed BAL in airways of normal and asthmatic subjects before and after challenges with bradykinin, aerosolized through a bronchoscope. They observed an increase in fibrinogen levels in the BAL fluid from both groups, thereby suggesting bradykinin-induced microvascular leakage [39].

### Angiogenesis

Unlike the pulmonary circulation, the bronchial vessels are known to have a considerable ability to proliferate during several pathological conditions. Leonardo da Vinci is generally regarded as the first person to observe, around a cavitatory lesion in human lung, the presence of vascular phenomena, that could be interpreted as angiogenetic processes [40]. In the last two decades, many reports have documented angiogenesis of bronchial vessels in response to a wide variety of stimuli, including chronic airway inflammation. Angiogenesis can be defined as the formation of new vessels by sprouting from pre-existing vessels [41]. With a broad meaning, lengthening and enlargement of existing vessels are also considered to be angiogenesis, whereby the vessels take a more tortuous course.

To explore and quantify the bronchial microvasculature, different methodological approaches are possible. Biopsy specimens from post-mortem resections provide considerable amounts of tissue with good clinical characterisation [11,21]. Similarly, lung resection studies allow for harvesting of pulmonary tissue in reasonable quantities, even if the tying procedure at the resection margin could influence the bronchial vascular congestion [21]. Fiberoptic bronchoscopy offers the possibility of obtaining repeated samples from the same patients, with varying disease severity, and evaluating pharmacological effects [42]. Finally, high magnification video bronchoscopy, a more recent and less invasive technique, is also useful to study the bronchial microvascularity in asthma and COPD [43,44].

Immunohistochemical analysis represents the gold standard approach to quantify bronchial microvascularity. The quantification of bronchial vessels generally includes the number of vessels per square millimetre of area analyzed, the vascular area occupied by the vessels, expressed as percentage of the total area evaluated, and the mean vessel size, estimated by dividing the total vascular area by the total number of vessels [12,17,42,45,46].

Two studies used the monoclonal antibody against factor VIII and analyzed the entire submucosa, finding both an increase vascular areas and vessel dimensions in asthmatic patients in comparison to healthy controls [11,21]. Furthermore, to obtain a better identification of the bronchial microvascularity, some groups of authors used the monoclonal antibody against collagen type IV [12,42,45-47], and performed the quantification in the supepithelial lamina propria [12,42,45,47] or in the entire submucosa [46]. Except for the Orsida study [45], which showed that asthmatics had only an increase in vascular area when compared to healthy controls, the other studies found an increase both in vessel number and in vascular area [12,42,46]. Salvato stained his sections with a combination of haematoxylin-eosin, Masson trichrome, PAS, alcian blue-PAS and orcein, and evaluated microvessels in the supepithelial lamina propria, showing an increase in vessel number and vascular area [13]. Moreover, some studies used the monoclonal antibody anti-CD31 [17,47,48], apparently more vessel-specific, but further investigation is required to obtain a better technique to measure the various aspects of angiogenesis [49].

In asthmatic patients, even with mild to moderate disease, a significant increase in the number of vessels and/or percent vascular area, as well as an increased average capillary dimension, can be observed in comparison to healthy controls [12,13,17,42,45-47]. Furthermore, a relationship between increased bronchial microvascularity and the severity of asthmatic disease was found [13,17,50]. Finally, the vascular component of airway remodelling in asthma does not appear related to the duration of the disease, given that it can be detectable even in asthmatic children [48].

**Figure 2:**
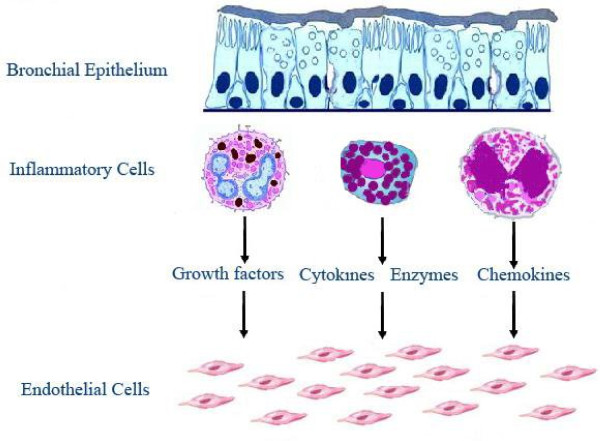
**Schematic picture of the angiogenic processes, indicating the activation and proliferation of endothelial cells**.

Angiogenesis should be considered to be a complex multiphase process, potentially involving a great number of growth factors, cytokines, chemokines, enzymes and other factors (Table 2 and Figure 2). The specific role of each molecule has not been clearly defined, even if VEGF is considered to be the most important angiogenic factor in asthma [51]. Many biopsy studies [47,52-54], as well as reports conducted on induced sputum [28-31], have observed higher VEGF levels in asthmatic airways when compared to those of healthy controls. In particular, immunohistochemical studies demonstrated close relationships between VEGF expression and vascularity [47,52-54]. Hoshino et al [53] found an increased staining of *m*RNA for VEGF receptors (R1 and R2) on bronchial endothelium in asthmatic patients versus normal subjects. Feltis et al. [47] showed a relationship between VEGF and VEGF-R2 expression in asthma and between VEGF and VEGF-R1 in controls.

It is of note that VEGF-R2 is the major mediator of the mitogenic, angiogenic and permeability-enhancing effects of VEGF, while VEGF-R1 has been suggested to act as a modulating decoy to VEGF-R2, thereby inhibiting VEGF-VEGF-R2 binding [55]. Therefore, it is plausible that VEGF-R2 is actively engaged in enhanced VEGF activity in asthma, possibly contributing to an increase in VEGF-induced microvascular remodelling [47]. Interestingly, Feltis et al [47] observed cystic structures within the vessel walls, that they termed angiogenic sprouts. The number of sprouts was markedly increased in asthmatics, and the increase in sprouts per vessel was positively related to the VEGF expression [47].

The angiopoietin family can play a role in vascular remodelling of asthmatic airways. Angiopoietin-1 (Ang-1) is known to stabilize new vessels, while angiopoietin-2 (Ang-2), in the presence of VEGF, acts as an Ang-1 antagonist, making vessels unstable and promoting vessel sprouting [47]. In contrast, endostatin is known to be a strong endogenous inhibitor of angiogenesis [56]. Interestingly, an imbalance between VEGF and endostatin levels was found in induced sputum from asthmatic patients [28]. In biopsies from asthmatic patients, Hoshino et al [52], showed higher expression of basic fibroblast growth factor (bFGF) and angiogenin, with significant correlations between the vascular area and the number of angiogenic factor-positive cells within the airways.

Metalloproteinases (MMPs) can also play a role in the angiogenic processes. MMPs are a large family of zinc- and calcium-dependent peptidases, which are able to degrade most components of tissue extracellular matrix. This function represents an essential requirement to permit cell migration in tissue, lengthening of existing vessels, and sprouting and formation of new vessels. Lee et al. [57] found that levels of VEGF and MMP-9 were significantly higher in the sputum of patients with asthma than in healthy control subjects, as well as a significant correlation between the levels of VEGF and MMP-9 was present. Moreover, administration of VEGF receptor inhibitors reduced the pathophysiological signs of asthma and decreased the expression of MMP-9 [57].

Many inflammatory cells are probably involved as angiogenic growth factor sources in the asthmatic airways. Mast cells are known to be one of the most important sources of proangiogenic factors [58]. Mast cells can secrete several mediators involved in angiogenesis, such as VEGF, bFGF, TGFβ, MMPs, histamine, lipid-derived mediators, chemokines (IL-8 in particular), cytokines, and proteases (Table 3). Co-localization studies revealed that both tryptase-positive mast cells and chymase-positive mast cells can play a role in the vascular component of airway remodelling in asthma, through induction of VEGF [54,59]. Other studies showed that in asthmatic patients, eosinophils, macrophages and CD34-positive cells can be involved as angiogenic growth factor sources [52,53].

**Table 3:** Major mast cells mediators, many of which have angiogenic activity

Mediators	Histamine, Tryptase, Chymase, Heparine, Carboxypeptidase A, MMP-2, MMP-9
**Cytokines**	IL-3, IL-4, IL-5, IL-6, IL-8, IL-9, IL-10, IL-13, TNFα

**Chemokines**	RANTES, Eotaxin, MCP-1, MCP-3, MCP-4, IL-8

**Growth Factors**	VEGF, bFGF, TGFβ, GM-CSF, PDGF, PAF

**Lipid-derived compounds**	PGD_2_, LTB_4_, LTC_4_, LTD_4_, LTE_4_

## COPD

The microvascular changes in the bronchial mucosa of COPD patients have recently aroused researchers' interest. Bosken et al [60] reported that the airways of patients with COPD were thicker than those of controls. Performing a morphometric analysis, they observed that muscle, epithelium, and connective tissue were all increased in the obstructed patients, and suggested that airway wall thickening contributes to airway narrowing. Kuwano et al. [21] conducted the first study on bronchial vascularity including COPD patients. They observed no difference in vascular area and number of vessels between COPD and controls, so the airway wall thickening in COPD patients was ascribed to an increase in airway smooth muscle. Notably, the lack of difference in vascularity between COPD and controls in the Kuwano study was probably due to the use of Factor VIII, which is not the gold standard to outline vessels [49].

More recently, Tanaka et al [43] assessed the airway vascularity in patients with asthma and COPD using a high-magnification bronchovideoscope and did not find any difference between COPD patients and controls [43]. A drawback of this technique is the detection limit of the bronchovideoscope and the incapacity to detect small vessels, with a size approximately less than 300 μ^2^.

**Figure 3:**
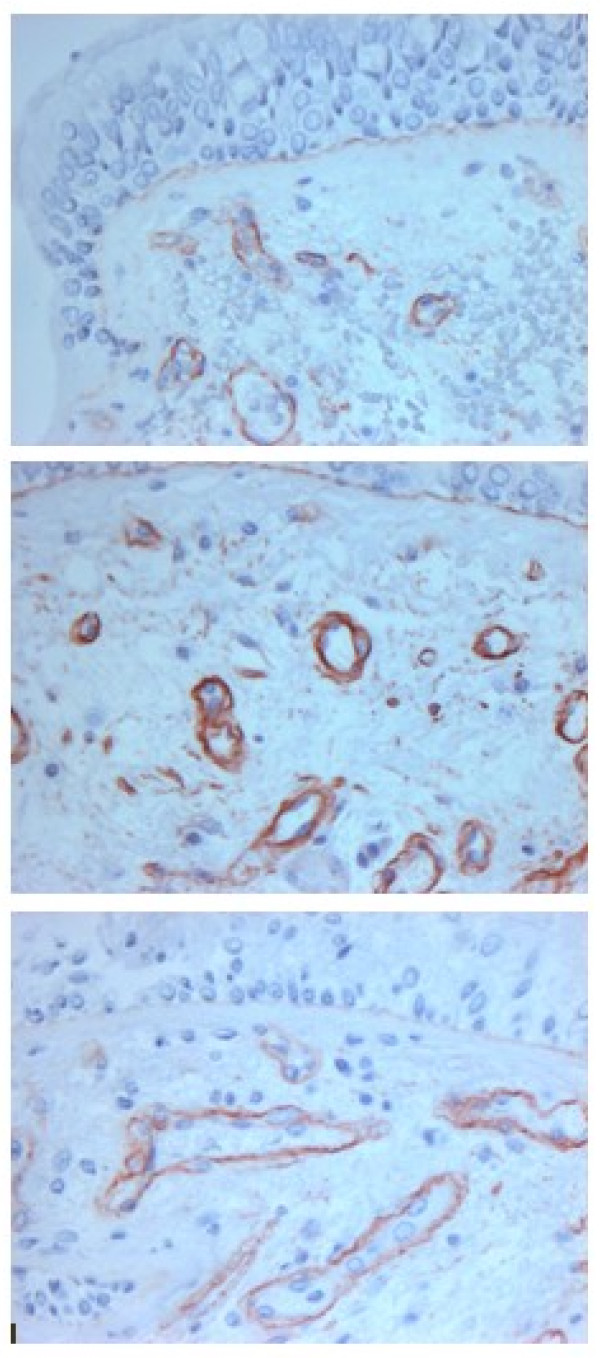
**Microphotographs from a normal subject (*upper panel*), asthmatic patient (*middle panel*) and COPD patient (*lower panel*) showing bronchial mucosa stained with antibody directed against Collagen IV to outline vessels. Original magnification × 400**.

Using immunohistochemistry and staining vessels with anti-CD31 monoclonal antibodies, Hashimoto et al. [17] showed that the number of vessels in the medium and small airways in asthmatic patients was increased, compared to those in COPD patients and controls. Furthermore, the vascular area was significantly increased in the medium airways in asthmatics and in the small airways in COPD patients, as compared to controls. Calabrese et al. [18] performed an immunohistochemical study on bronchial biopsies taken from the central airways of smokers with and without obstruction, using monoclonal antibodies against collagen type IV to outline vessels. They observed an increase both in vascular area and number of vessels in current smokers with COPD and in symptomatic smokers with normal lung function, when compared to healthy non-smokers [18]. In the central airways of clinically stable patients with COPD who were not current smokers, we could demonstrate a higher vascular area, but not an increase in the number of vessels, when compared to control subjects [61]. In spite of the differences in methods and patient selection criteria, all these studies [17,18,61] consistently found an increased vascular area in the airways of patients with COPD (Figure 3).

Interestingly, in a heterogeneous group of COPD patients with different comorbidities, such as lung cancer, bronchiectasis, lung transplantation, and chronic lung abscess, Polosukhin et al [62] found that reticular basement membrane thickness was increased and the subepithelial microvascular bed was reduced in association with progression from normal epithelium to squamous metaplasia. Recently, a group actively researching this area provided preliminary data which showed the presence of splitting and fragmentation of the reticular basement membrane associated with altered distribution of vascularity between the reticular basement membrane and the lamina propria in COPD patients and smokers as compared to controls [63,64].

Like in asthma, VEGF is implicated in the mechanisms of bronchial vascular remodelling in COPD. Kanazawa et al. [65] showed increased VEGF levels in induced sputum from patients with chronic bronchitis and asthma, and decreased levels from patients with emphysema, as compared to controls. Moreover, VEGF levels were negatively related to lung function in chronic bronchitis, but positively in emphysema, suggesting different actions in these two COPD subtypes [65]. By analysing the bronchial expression of VEGF and its receptors, Kranenburg et al. [66] showed that COPD was associated with increased expression of VEGF in the bronchial, bronchiolar and alveolar epithelium, in macrophages as well as in vascular and airway smooth muscle cells. More recently, Calabrese et al. [18] found an association between increased bronchial vascularity and both a higher cellular expression of VEGF and a vascular expression of α5β3 integrin. Interestingly, α5β3 integrin is an adhesion molecule that is upregulated in new vessel proliferation in response to angiogenic stimuli, while it is not expressed or only at low levels in resting endothelium [67].

An increased expression of FGF-1 and FGF-2 was found by Kranenburg et al. [68] in the bronchial epithelium of COPD patients. Additionally, FGF receptor-1 (FGFR-1) was detected in bronchial epithelial and airway smooth muscle cells and in the endothelium of bronchial vessels. Interestingly, a positive correlation between FGF-1 expression and pack-years was found, indicating that the degree of pulmonary FGF-1 expression may be related to the amount of airway exposure to smoke [68]. The extracellular matrix proteins may play a role in the remodelling of airways and blood vessels in COPD. In COPD patients, as compared to non-COPD patients an increased staining for fibronectin in the neointima, for collagen type IV and laminin in the medial layer, and for collagen type III in the adventitial layer of bronchial vessel walls was observed [69]. Polosukhin et al. [62] showed that in patients with COPD the percentage of HIF-1α positive epithelial cells significantly increased with reduction in blood vessel number, thickness of the reticular basement membrane and increased epithelial height. Other growth factors, involved in vascular changes during chronic inflammatory or neoplastic processes, such as EGF, IGF, PDGF and HGF [70] may also be involved in the vascular component of the bronchial remodelling in COPD, however experimental data are lacking in this area. Similarly, MMPs could also play a role in angiogenesis in COPD, as well as in asthma, however, this area of research has yet to be investigated.

Likely analogous to COPD, but much more rapid in onset, vascular changes may occur after lung transplantation. As reported by Walters and co-workers [9], over recent years attention has been given to airway inflammation and remodelling post lung transplantation. The pathologic airway changes observed show the characteristics of bronchiolitis obliterans syndrome. Angiogenic remodelling seems to occur early and it is not related to airflow limitation. Vessel number and vascular area are higher than in controls, and they are probably related to IL-8 and other C-X-C chemokines rather than VEGF levels [9].

## Significance of the vascular component of airway remodelling

Bronchial microvasculature changes may result in airway wall thickening and in the reduction of the lumenal area. Several studies on bronchial vascular remodelling in asthma and COPD showed significant correlations between morphological or biological data (number of vessels, vascular area, expression of angiogenic factors) and lung function parameters, such as FEV_1 _[17,28,30,45,46,52,53,66,68], FEV_1_/FVC [29,68] and airway hyperresponsiveness [29,36,45,46]. The functional effects of vascular remodelling may be amplified by pre-existing airway wall architecture modifications, such as bronchial mucosal thickening, cellular infiltration, collagen deposition and bronchial smooth muscle changes [71,72].

In functional terms, the airway wall can be considered as the sum of three distinct layers and the thickening of each can have separate effects [72]. The thickening of the inner airway wall layer (epithelium, lamina reticularis, and loose connective tissue between the lamina reticularis and the airway smooth muscle (ASM) layer) can amplify the effect of ASM shortening; the thickening of the outer (or adventitial) layer could decrease the static and dynamic loads on the ASM; and an increase in the ASM layer thickness can increase the strength of the muscle. In addition, the remodelling of the connective tissue in the smooth muscle compartment could increase or decrease the amount of radial constraint provided to the ASM. Finally, thickening and fibrous connective tissue deposition in all layers could decrease airway distensibility and allow ASM adaptation in shorter lengths [72].

Hypertrophy and hyperplasia of mucous glandular structures and loss of alveolar attachments are considered to be the main structural changes of airway remodelling in COPD and they may play a crucial role in the functional effects of airway remodelling (Table 4) [73]. However, in COPD patients the exact explanation for the link between structural airway changes, with particular reference to the vascular component, and the functional and clinical consequences are not yet clearly defined and further studies are needed.

**Table 4:** Main structural changes in airway remodelling and respective functional effects in asthma and COPD

Structural changes	Functional effects	Asthma	COPD
**Vascular remodelling **with inner airway wall thickening	Decreased baseline airway calibre and amplification of airway smooth muscle shortening	+++	+

Hypertrophy and hyperplasia of airway smooth muscle	Increased smooth muscle strength and airway hyperresponsiveness	+++	+

Connective tissue deposition	Increased airway smooth muscle radial constraint	+++	+

Thickening and fibrosis of all layers	Decreased airway distensibility and reduced effectiveness of bronchodilators	++	+

Hypertrophy and hyperplasia of mucus gland	Decreased lumen calibre and amplification of airway smooth muscle shortening	+	+++

Loss of alveolar attachments	Predisposition to expiratory closure and collapse	-	+++

## Bronchial vascular remodelling and pharmacological modulation

The bulk of the literature regarding the pharmacological effects on the vascular component of airway remodelling has been obtained from studies in asthmatic patients. The efficacy of anti-asthma drugs on vascular remodelling is schematically presented in Table 5. Inhaled corticosteroids are the only treatment able to positively affect all three main aspects of the vascular component of airway remodelling: vasodilatation, increased microvascular permeability and angiogenesis. Several studies on asthmatic airways indicate that high doses of inhaled corticosteroids (approximately a daily dose of BDP and FP ≥ 800 μg) may reverse the increased vascularity [28,29,42,45,46,54], while there is no consensus on the duration of treatment. Notably, this important effect seems to be especially mediated by a reduced expression of VEGF by inflammatory cells [28-30,54,74].

**Table 5:** The efficacy of anti-asthma drugs on aspects of the vascular components of airway remodelling.

	Corticosteroids	LABA	LTRAs
**Microvascular leakage**	**+++**	++	+

**Vasodilatation**	+++	-	+

**Angiogenesis**	+++	+	-

+++ = highly effective; ++ = effective; + = moderately effective; - = ineffective

*N.B*.: due to the lack of data regarding theophylline, we excluded it from the table

Less experimental evidence is available on the actions of long-acting β2 agonists (LABAs) and leukotriene receptor antagonists (LTRAs) on decreasing the vascular component of airway remodelling. Three months of treatment with salmeterol, in a placebo-controlled study, showed a significant decrease in the vascularity in the lamina propria of asthmatics [75]. This may have been caused by reduced levels of the angiogenic cytokine IL-8 following salmeterol treatment [76]. Moreover, among anti-leukotrienes, montelukast has proven to have an acute effect on decreasing airway mucosal blood flow, similar to inhaled steroids [77].

There is a dearth of published data regarding the effect of current therapies for COPD on bronchial microvascularity. Recently, in a cross-sectional study on COPD patients, we found that, as compared to untreated patients, treated patients with long-term high doses of beclomethasone showed lower values of vascular area and lower expression of VEGF, bFGF, and TGFβ [61]. Further prospective studies are needed to confirm this finding and to assess the possible role of bronchodilators on the bronchial microvascularity in COPD.

Finally, several inhibitors of angiogenesis have been studied in vitro and consequently progressed to clinical studies. Some of them have shown promising results in lung cancer, but these molecules have not yet been investigated in chronic airway disease [78]. In the future, these compounds, especially the therapeutic agents that antagonize the effect of VEGF and/or prevent its production could represent a novel approach for positively acting on bronchial microvascular changes in asthma and COPD (Table 6).

**Table 6:** Drugs potentially active on airway vascular remodelling

Name	Type of compound	Mode of Action
**Drugs active on VEGF** Bevacizumab	Humanized IgG1 monoclonal antibody against VEGF	Neutralization of VEGF
		
VEGF Trap	Engineered soluble receptor	Prevention of ligand binding
		
Sorafenib, Sunitinib	Multitargeted receptor tyrosine kinase inhibitors	Inhibition of signal transduction and transcription
		
Neovastat	Multifunctional agent obtained from dogfish cartilage	Interference with VEGFR2 Induction of EC apoptosis Inhibition of MMP activities
		
**Drugs active on FGF** SU6668	Competitive inhibitor of FGFR1, Flk-1/KDR, PDGFRβ via receptor tyrosine kinase	Inhibition of signal transduction and transcription
		
**Drugs active on TGFβ** SR2F	Antagonist of the soluble receptor:Fc fusion protein class	Neutralization of TGFβ
		
**Drugs active on MMPs** AZ11557272	MMP-9/MMP-12 inhibitor	Inhibition of collagen and elastin destruction

EC = endothelial cell, FGF = fibroblast growth factor, MMP = metalloproteinase, PDGF = platelet-derived growth factor, TGF = transforming growth factor, VEGF = vascular endothelial growth factor

## Conclusions

In the last two decades there has been an increasing interest in the microvascular changes of bronchial airway mucosa during chronic inflammation, such as asthma and COPD. Up to now, the focus of researchers has been on asthma, while little work has been done in COPD. Moreover, the effects of smoking on bronchial microvascularity need better differentiation from the specific changes due to airflow obstruction.

Although evidence suggests that there are vessel changes in the bronchial wall in both asthma and COPD, there are distinct differences in detail between these situations. Angiogenesis seems to be a typical finding in asthma, while, in COPD, little evidence supports the view that vasodilatation is prevalent.

Microvascular changes in the bronchial airway mucosa are probably the consequence of the activities of many angiogenic factors, but VEGF seems to be crucially involved both in asthma and COPD, and different types of cells can play a role as the source of VEGF.

The clinical and functional relevance of the vascular remodelling remains to be determined both in asthma and COPD; even if some reports suggest that the vascular component of airway remodelling may contribute to worsening of airway function.

There are few data regarding the effects of current therapies on bronchial vascular remodelling. Some longitudinal studies were conducted in asthma, with biopsy quantification of vascular changes. These studies showed that inhaled corticosteroids could effectively act on vascular remodelling in asthmatic airways, partially reversing microvascular changes. Evidence from a cross-sectional study suggests also that long-term treatment with inhaled corticosteroids is associated with a reduction in airway microvascularity in COPD.

Better knowledge about angiogenic processes and their consequences in the airway wall both in asthma and especially COPD are urgently needed, as well as new therapeutic strategies for these conditions.

## Competing interests

All authors have no competing interest. There was no funding provided for this manuscript.

## Authors' contributions

All authors participated in drafting the manuscript. All authors read and approved the final version of the manuscript.

## References

[B1] Widdicombe J Why are the airways so vascular? Thorax 1993 48 290 295 10.1136/thx.48.3.290 8497832 PMC464376

[B2] Baile EM The anatomy and physiology of the bronchial circulation J Aerosol med 1996 9 1 6 10.1089/jam.1996.9.1 10160199

[B3] Charan NB Baile EM Pare PD Bronchial vascular congestion and angiogenesis Eur Respir J 1997 10 1173 1180 10.1183/09031936.97.10051173 9163664

[B4] John GW Willium WB, Stephen TH Microvascular anatomy of the airways Asthma and rhinitis 2000 2 Malden, MA: Blackwell Science 721 731

[B5] Deffebach ME Lung mechanical effects on the bronchial circulation Eur Respir J Suppl 1990 12 586s 590s 2076152

[B6] Lung MAKY Wang JCC Cheng KK Bronchial circulation: an auto-perfusion method for assessing its vasomotor acitivity and the study of alpha- and beta-adrenoceptors in the bronchial artery Life Sci 1976 19 577 580 10.1016/0024-3205(76)90239-3 957893

[B7] Lieckens S Angiogenesis: regulators and clinical applications Biochem Pharmacol 2001 61 253 270 10.1016/S0006-2952(00)00529-3 11172729

[B8] Deffebach ME Charan NB Lakshminarayan S Butler J The bronchial circulation. Small, but a vital attribute of the lung Am Rev Respir Dis 1987 135 463 480 3544986 10.1164/arrd.1987.135.2.463

[B9] Walters EH Reid D Soltani A Ward C Angiogenesis: a potentially critical part of remodelling in chronic airway diseases? Pharmacol Ther 2008 118 128 137 10.1016/j.pharmthera.2008.01.007 18358536

[B10] McDonald DM Angiogenesis and remodeling of airway vasculature in chronic inflammation Am J Respir Crit Care Med 2001 164 S39 S45 11734465 10.1164/ajrccm.164.supplement_2.2106065

[B11] Carroll NG Cooke C James AL Bronchial blood vessels dimensions in asthma Am J Respir Crit Care Med 1997 155 689 695 9032214 10.1164/ajrccm.155.2.9032214

[B12] Li X Wilson JW Increased vascularity of the bronchial mucosa in mild asthma Am J Respir Crit Care Med 1997 156 229 233 9230753 10.1164/ajrccm.156.1.9607066

[B13] Salvato G Quantitative and morphological analysis of the vascular bed in bronchial biopsy specimens from asthmatic and non-asthmatic subjects Thorax 2001 56 902 906 10.1136/thorax.56.12.902 11713351 PMC1745985

[B14] Wilson JW Hii S The importance of the airway microvasculature in asthma Curr Opin Allergy Clin Immunol 2006 6 51 55 10.1097/01.all.0000200505.54425.47 16505612

[B15] Chetta A Zanini A Torre O Olivieri D Vascular remodelling and angiogenesis in asthma: morphological aspects and pharmacological modulation Inflamm Allergy Drug Targets 2007 1 41 45 10.2174/187152807780077273 17352687

[B16] Bergeron C Boulet LP Structural changes in airway diseases Chest 2006 129 1068 1087 10.1378/chest.129.4.1068 16608960

[B17] Hashimoto M Tanaka H Abe S Quantitative analysis of bronchial wall vascularity in the medium and small airways of patients with asthma and COPD Chest 2005 127 965 972 10.1378/chest.127.3.965 15764783

[B18] Calabrese C Bocchino V Vatrella A Marzo C Guarino C Mascitti S Tranfa CME Cazzola M Micheli P Caputi M Marsico SA Evidence of angiogenesis in bronchial biopsies of smokers with and without airway obstruction Resp Med 2006 100 1415 1422 10.1016/j.rmed.2005.11.009 16497496

[B19] Dunnill MS The pathology of asthma with special reference to changes in the bronchial mucosa J Clin Pathol 1960 13 27 33 10.1136/jcp.13.1.27 13818688 PMC479992

[B20] Dunnill MS Massarella GR Anderson JA A comparison of the quantitative anatomy of the bronchi in normal subjects, in status asthmaticus, in chronic bronchitis and in emphysema Thorax 1969 24 176 179 10.1136/thx.24.2.176 5821620 PMC471937

[B21] Kuwano K Bosken CH Paré PD Bai TR Wiggs BR Hogg JC Small airways dimensions in asthma and in chronic obstructive pulmonary disease Am Rev Respir Dis 1993 148 1220 1225 8239157 10.1164/ajrccm/148.5.1220

[B22] Kumar SD Emery MJ Atkins ND Danta I Wanner A Airway mucosal blood flow in bronchial asthma Am J Respir Crit Care Med 1998 158 153 156 9655722 10.1164/ajrccm.158.1.9712141

[B23] Bailey SR Boustany S Burgess JK Hirst SJ Sharma HS Simcock DE Suravaram PR Weckmann M Airway vascular reactivity and vascularisation in human chronic airway disease Pulm Pharmacol Ther 2009 22 417 425 10.1016/j.pupt.2009.04.007 19409504

[B24] Charan NB Johnson SR Lakshminarayan S Thompson WH Carvalho P Nitric oxide and beta-adrenergic agonist-induced bronchial arterial vasodilation J Appl Physiol 1997 82 686 692 9049753 10.1152/jappl.1997.82.2.686

[B25] Paredi P Kharitonov SA Barnes PJ Correlation of exhaled breath temperature with bronchial flow in asthma Respir Res 2005 6 15 10.1186/1465-9921-6-15 15705206 PMC553993

[B26] Chung KF Rogers DF Barnes PJ Evans TW The role of increased airway microvascular permeability and plasma exudation in asthma Eur Respir J 1990 3 329 337 2187708

[B27] McDonald DM The ultrastructure and permeability of tracheobronchial blood vessels in health and disease Eur Respir J 1990 12 Suppl 572 585 2076151

[B28] Asai K Kanazawa H Kamoi H Shiraishi S Hirata K Yoshikawa J Increased levels of vascular endothelial growth factor in induced sputum in asthmatic patients Clin Exp Allergy 2003 33 595 599 10.1046/j.1365-2222.2003.01576.x 12752587

[B29] Kanazawa H Nomura S Yoshikawa J Role of microvascular permeability on physiologic differences in asthma and eosinophilic bronchitis Am J Respir Crit Care Med 2004 169 1125 1130 10.1164/rccm.200401-123OC 15044203

[B30] Kanazawa H Hirata H Yoshikawa J Involvement of vascular endothelial growth factor in exercise induced bronchoconstriction in asthmatic patients Thorax 2002 57 885 888 10.1136/thorax.57.10.885 12324676 PMC1746203

[B31] Kanazawa H Nomura S Asai K Roles of angiopoietin-1 and angiopoietin-2 on airway microvascular permeability in asthmatic patients Chest 2007 131 1035 1041 10.1378/chest.06-2758 17426207

[B32] Meerschaert J Becky Kelly EA Mosher DF Busse WW Jarjour NN Segmental antigen challenge increases fibronectin in bronchoalveolar lavage fluid Am J Respir Crit Care Med 1999 159 619 625 9927382 10.1164/ajrccm.159.2.9806053

[B33] Svensson C Grönneberg R Andersson M Alkner U Andersson O Billing B Gilljam H Greiff L Persson CGA Allergen challenge-induced entry of alpha 2-macroglobulin and tryptase into human nasal and bronchial airways J Allergy Clin Immunol 1995 96 239 246 10.1016/S0091-6749(95)70013-7 7543503

[B34] Goldie RG Pedersen KE Mechanisms of increased airway microvascular permeability: role in airway inflammation and obstruction Clin Exp Pharmacol Physiol 1995 22 387 396 10.1111/j.1440-1681.1995.tb02028.x 8582087

[B35] James AL Pare PD Hogg JC The mechanics of airway narrowing in asthma Am Rev Respir Dis 1989 139 242 246 2912345 10.1164/ajrccm/139.1.242

[B36] Otani K Kanazawa H Fujiwara H Hirata K Fujimoto S Yoshikawa J Determinants of the severity of exercise-induced bronchoconstriction in patients with asthma J Asthma 2004 41 271 278 10.1081/JAS-120026083 15260459

[B37] Roberts WG Palade GE Increased microvascular permeability and endothelial fenestration induced by vascular endothelial growth factor J Cell Sci 1995 108 2369 2379 7673356 10.1242/jcs.108.6.2369

[B38] Greiff L Wollmer P Andersson M Svensson C Persson CGA Effects of formoterol on histamine induced plasma exudation in induced sputum from normal subjects Thorax 1998 53 1010 1013 10.1136/thx.53.12.1010 10195069 PMC1745146

[B39] Berman AR Liu MC Wagner EM Proud D Dissociation of bradykinin-induced plasma exudation and reactivity in the peripheral airways Am J Respir Crit Care Med 1996 154 418 423 8756816 10.1164/ajrccm.154.2.8756816

[B40] Cudkowicz L Leonardo da Vinci and the bronchial circulation Br J Dis Chest 1953 47 649 670 13019101

[B41] Folkman J Clinical applications of research on angiogenesis New Engl J Med 1995 333 1951 1957 10.1056/NEJM199512283332608 7491141

[B42] Chetta A Zanini A Foresi A Del Donno M Castagnaro A D'Ippolito R Baraldo S Testi R Saetta M Olivieri D Vascular component of airway remodelling in asthma is reduced by high dose of fluticasone Am J Respir Crit Care Med 2003 167 751 757 10.1164/rccm.200207-710OC 12468439

[B43] Tanaka H Yamada G Saikai T Hashimoto M Tanaka S Suzuki K Fujii M Takahashi H Abe S Increased airway vascularity in newly diagnosed asthma using a high-magnification bronchovideoscope Am J Respir Crit Care Med 2003 168 1495 1499 10.1164/rccm.200306-727OC 14512267

[B44] Yamada G Takahashi H Shijubo N Itoh T Abe S Subepithelial microvasculature in large airways observed by high-magnification bronchovideoscope Chest 2005 128 876 880 10.1378/chest.128.2.876 16100181

[B45] Orsida BE Li X Hickey B Thien F Wilson JW Walters EH Vascularity in asthmatic airways: relation to inhaled steroid dose Thorax 1999 54 289 295 10.1136/thx.54.4.289 10092688 PMC1745476

[B46] Hoshino M Takahashi M Takai Y Sim J Aoike N Inhaled corticosteroids decrease vascularity of the bronchial mucosa in patients with asthma Clin Exp Allergy 2001 31 722 730 10.1046/j.1365-2222.2001.01071.x 11422131

[B47] Feltis NB Wignarajah D Zheng L Ward C Reid D Harding R Walters EH Increased vascular endothelial growth factor and receptors Am J Respir Crit Care Med 2006 173 1201 1207 10.1164/rccm.200507-1105OC 16528018

[B48] Barbato A Turato G Baraldo S Bazzan E Calabrese F Panizzolo C Zanin ME Zuin R Maestrelli P Fabbri LM Saetta M Epithelial damage and angiogenesis in the airways of children with asthma Am J Respir Crit Care Med 2006 174 975 981 10.1164/rccm.200602-189OC 16917118

[B49] Vermeulen PB Gasparini G Fox SB Colpaert C Marson LP Gion M Beliën JAM de Waal RMW Van Marck E Magnani E Weidner N Harris AL Dirix LY Second international consensus on the methodology and criteria of evaluation of angiogenesis quantification in solid human tumours Eur J Cancer 2002 38 1564 1579 10.1016/S0959-8049(02)00094-1 12142044

[B50] Vrugt B Wilson S Bron A Holgate ST Djukanovic R Aalbers R Bronchial angiogenesis in severe glucocorticoid-dependent asthma Eur Respir J 2000 15 1014 1021 10.1034/j.1399-3003.2000.01507.x 10885418

[B51] Walters EH Soltani A Reid DW Ward C Vascular remodelling in asthma Curr Opinion Allergy Clin Immunol 2008 8 39 43 10.1097/ACI.0b013e3282f42696 18188016

[B52] Hoshino M Takahashi M Aoike N Expression of vascular endothelial growth factor, basic fibroblast growth factor, and angiogenin immunoreactivity in asthmatic airways and its relationship to angiogenesis J Allergy Clin Immunol 2001 107 295 301 10.1067/mai.2001.111928 11174196

[B53] Hoshino M Nakamura Y Hamid Q Gene expression of vascular endothelial growth factor and its receptors and angiogenesis in bronchial asthma J Allergy Clin Immunol 2001 107 1034 1038 10.1067/mai.2001.115626 11398081

[B54] Chetta A Zanini A Foresi A D'Ippolito R Tipa A Castagnaro A Baraldo S Neri M Saetta M Olivieri D Vascular endothelial growth factor and bronchial wall remodelling in asthma Clin Exp Allergy 2005 35 1437 1442 10.1111/j.1365-2222.2005.02360.x 16297139

[B55] Ferrara N Gerber HP LeCouter J The biology of VEGF and its receptors Nat Med 2003 9 669 676 10.1038/nm0603-669 12778165

[B56] O'Reilly MS Bohem T Shing Y Fukai N Vasios G Lane WS Flynn E Birkhead JR Olsen BR Folkman J Endostatin: an endogenous inhibitor of angiogenesis and tumor growth Cell 1997 88 277 285 10.1016/S0092-8674(00)81848-6 9008168

[B57] Lee KS Min KH Kim SR Park SJ Park HS Jin GJ Lee YC Vascular endothelial growth factor modulates metalloproteinase-9 expression in asthma Am J Respir Crit Care Med 2006 174 161 170 10.1164/rccm.200510-1558OC 16645174

[B58] Norrby K Mast cells and angiogenesis APMIS 2002 110 355 371 10.1034/j.1600-0463.2002.100501.x 12076253

[B59] Zanini A Chetta A Saetta M Baraldo S D'Ippolito R Castagnaro A Neri M Olivieri D Chymase-positive mast cells play a role in the vascular component of airway remodelling in asthma J Allergy Clin Immunol 2007 120 329 333 10.1016/j.jaci.2007.04.021 17559912

[B60] Bosken CH Wiggs BR Paré PD Hogg JC Small airway dimensions in smokers with obstruction to airflow Am Rev Respir Dis 1990 142 563 570 2389908 10.1164/ajrccm/142.3.563

[B61] Zanini A Chetta A Nicolini G Castagnetti C D'Ippolito R Neri M Olivieri D Vascular remodelling and inhaled beclomethasone dipropionate in COPD Thorax 2009 64 1019 1024 10.1136/thx.2009.114629 19736178

[B62] Polosukhin VV Lawson WE Milstone AP Egunova SM Kulipanov AG Tchuvakin SG Massion PP Balckwell TS Association of progressive structural changes in the bronchial epithelium with subepithelial fibrous remodeling: a potential role for hypoxia Virchows Arch 2007 451 793 803 10.1007/s00428-007-0469-5 17674038

[B63] Soltani A Sohal S Reid D Muller HK Weston S Wood-Baker R Walters EH Inhaled corticosteroids in airway remodelling in COPD Thorax 2009 64 Suppl 4 A55

[B64] Soltani A Reid D Sohal S Muller HK Weston S Wood-Baker R Walters EH Vascular and basement membrane remodelling in smokers and COPD Eur Respir J 2009 Suppl 53 485 10.1186/1465-9921-11-105 PMC2918561 20670454

[B65] Kanzawa H Asai K Hirata K Yoshikawa J Possible effects of vascular endothelial growth factor in the pathogenesis of chronic obstructive pulmonary disease Am J Med 2003 114 354 358 10.1016/S0002-9343(02)01562-0 12714123

[B66] Kranenburg AR de Boer WI Alagappan VKT Sterk PJ Sharma HS Enhanced bronchial expression of vascular endothelial growth factor and receptors (Flk-1 and Flt-1) in patients with chronic obstructive pulmonary disease Thorax 2005 60 106 113 10.1136/thx.2004.023986 15681497 PMC1747292

[B67] Soldi R Mitola S Strasly M Defilippi P tarone G Bussolino F Role of alphavbeta3 integrin in the activation of vascular endothelial growth factor receptor-2 EMBO J 1999 18 882 892 10.1093/emboj/18.4.882 10022831 PMC1171181

[B68] Kranenburg AR Willems-Widyastuti A Mooi WJ Saxena PR Sterk PJ de Boer WI Sharma HS Chronic obstructive pulmonary disease is associated with enhanced bronchial expression of FGF-1, FGF-2 and FGFR-1 J Pathol 2005 206 28 38 10.1002/path.1748 15772985

[B69] Kranenburg AR Willems-Widyastuti A Mooi WJ Sterk PJ Alagappan VKT de Boer WI Sharma HS Enhanced bronchial expression of extracellular matrix proteins in chronic obstructive pulmonary disease Am J Clin Pathol 2006 126 725 735 10.1309/JC477FAEL1YKV54W 17111536

[B70] Maruotti N Cantatore FP Crivellato E Vacca A Ribatti D Angiogenesis in rheumatoid arthritis Histol Histopathol 2006 21 557 566 16493585 10.14670/HH-21.557

[B71] Wilson JW Kotsimbos T Airway vascular remodelling in asthma Curr Allergy Asthma Rep 2003 3 153 158 10.1007/s11882-003-0028-3 12562555

[B72] Wang L McParland BE Paré PD The functional consequences of structural changes in airways Chest 2003 123 356S 362S 10.1378/chest.123.3_suppl.356S-a 12628973

[B73] Kim V Rogers TJ Criner GJ New concepts in the pathobiology of chronic obstructive pulmonary disease Proc Am Thorac Soc 2008 5 478 485 10.1513/pats.200802-014ET 18453359 PMC2645323

[B74] Feltis BN Wignarajah D Reid DW Ward C Harding R Walters EH Effects of inhaled fluticasone on angiogenesis and vascular endothelial growth factor in asthma Thorax 2007 62 314 319 10.1136/thx.2006.069229 17105777 PMC2092477

[B75] Orsida BE Ward C Li X Bish R Wilson J Thien F Walters EH Effect of a long-acting β2-agonist over three months on airway wall vascular remodelling in asthma Am J Respir Crit Care Med 2001 164 117 121 11435249 10.1164/ajrccm.164.1.2006003

[B76] Reid DW Ward C Wang N Zheng L Bish R Orsida BE Walters EH Possible anti-inflammatory effect of salmeterol against interleukin-8 and neutrophil activation in asthma in vivo Eur Respir J 2003 21 994 999 10.1183/09031936.03.00109702 12797494

[B77] Mendes ES Campos MA Hurtado A Wanner A Effect of montelukast and fluticasone propionate on airway mucosal blood flow in asthma Am J Respir Crit Care Med 2004 169 1131 1134 10.1164/rccm.200311-1544OC 15028562

[B78] Horn L Sandler AB Angiogenesis in the treatment of non-small cell lung cancer Proc Am Thorac Soc 2009 6 206 217 10.1513/pats.200807-066LC 19349490 PMC5820832

